# Enhancing integration of administrative databases in South Africa's HIV program: Validation of record linkage using non-representative gold standard

**DOI:** 10.23889/ijpds.v9i5.2899

**Published:** 2024-09-18

**Authors:** Evelyn Lauren, Dickman Gareta, Khumbo Shumba, Dorina Onoya, Jacob Bor

**Affiliations:** 1 Department of Biostatistics, Boston University School of Public Health; 2 Health Economics and Epidemiology Research Office; 3 Africa Health Research Institute; 4 Institute of Social and Preventive Medicine, University of Bern

## Introduction

Linked administrative data are widely used in epidemiology to capture patient data across multiple databases. Linkage error rates, critical to measure linkage performance, are rarely reported due to difficulty in obtaining representative gold standard. We propose a training and validation approach for linkage procedures that yield unbiased performance estimates even with a non-representative gold standard.

## Methods

We linked patient records from two non-deduplicated databases for HIV monitoring in South Africa, TIER.Net and NHLS laboratory database, using a network-based probabilistic linkage and deduplication approach. National IDs (gold standard) were available for a non-representative minority of records (10%). We calculated sensitivity (Sen, share of true matches identified by the algorithm) and positive predictive value (PPV, share of algorithm-identified matches that were true matches). We adjusted for bias due to informative missingness in National IDs using inverse probability weights to break the link between missingness and match probability.

## Results

111,755 record pairs were considered. National IDs were not missing completely at random. Match probabilities for National ID record pairs exhibited substantially less uncertainty (mid-range match probabilities), inflating Sen and PPV. Before bias correction, Sen and PPV were estimated at 97.0% and 97.8% respectively. After bias correction for missing National IDs, Sen and PPV were estimated at 95.7% and 96.6%. Failure to address this bias understated the overlinkage rate (100% - PPV) by 35% and the underlinkage rate (100% - Sen) by 30%.

## Conclusion

Failure to adjust for informative missingness in the gold standard may lead to biased validation metrics and over/underconfidence in linked data.

